# Risk of Childhood Cancers Associated with Residence in Agriculturally Intense Areas in the United States

**DOI:** 10.1289/ehp.9967

**Published:** 2008-01-10

**Authors:** Susan E. Carozza, Bo Li, Kai Elgethun, Ryan Whitworth

**Affiliations:** 1 Department of Epidemiology and Biostatistics, School of Rural Public Health, Texas A&M Health Science Center, College Station, Texas, USA; 2 National Center for Atmospheric Research, Boulder, Colorado, USA; 3 Department of Community and Environmental Health, Boise State University, Boise, Idaho, USA; 4 Centers for Health Promotion and Prevention Research, School of Public Health, University of Texas Health Science Center at Houston, Houston, Texas, USA

**Keywords:** agricultural pesticides, childhood cancers, farming, pediatric cancers

## Abstract

**Background:**

The potential for widespread exposure to agricultural pesticides through drift during application raises concerns about possible health effects to exposed children living in areas of high agricultural activity.

**Objectives:**

We evaluated whether residence in a county with greater agricultural activity was associated with risk of developing cancer in children < 15 years of age.

**Methods:**

Incidence data for U.S. children 0–14 years of age diagnosed with cancer between 1995 and 2001 were provided by member registries of the North American Association of Central Cancer Registries. We determined percent cropland for each county using agricultural census data, and used the overall study distribution to classify agriculturally intense counties. We estimated odds ratios and 95% confidence intervals for all ages and 5-year age groups for total cancers and selected cancer sites using logistic regression.

**Results:**

Our study results showed statistically significant increased risk estimates for many types of childhood cancers associated with residence at diagnosis in counties having a moderate to high level of agricultural activity, with a remarkably consistent dose–response effect seen for counties having ≥ 60% of the total county acreage devoted to farming. Risk for different cancers varied by type of crop.

**Conclusions:**

Although interpretation is limited by the ecologic design, in this study we were able to evaluate rarer childhood cancers across a diverse agricultural topography. The findings of this exploratory study support a continued interest in the possible impact of long-term, low-level pesticide exposure in communities located in agriculturally intense areas.

Increased incidence of certain cancers among farmers and workers employed in agricultural settings has been reported in a variety of epidemiologic studies, raising concerns about exposure to agricultural chemicals in general and agricultural pesticides in particular (Blair and Zahm 1991, 1995; Blair et al. 1993; De Roos et al. 2003). Agricultural pesticides routinely spread beyond the intended agricultural target area, with drift possible for miles depending on wind conditions and particle size (Tiefenbacher 1998; van den Berg et al. 1999). Because measurable amounts of agricultural pesticides have been reported in non-farming households and communities [Baker et al. 1996; Centers for Disease Control and Prevention (CDC) 2005; Koch et al. 2002; Shalat et al. 2003], it is probable that pesticide drift provides a mechanism by which exposure can occur not only to agricultural workers but also to their families and neighbors.

This potential for widespread exposure raises concerns about possible health effects in the offspring of women who are exposed to these pesticides during pregnancy and in very young children, a population whose age-related behaviors put them at particularly high risk of exposure. Children living in areas of high agricultural activity may be exposed to higher levels of pesticides than other children, through playing in nearby fields, increased opportunities for pesticides to be tracked into the home by various household members, and breast milk from exposed mothers (Eskenazi et al. 1999). In addition to having more opportunities for exposure, young children may prove to be particularly vulnerable to lower-dose exposures of pesticides with teratogenic and carcinogenic potential (Faustman et al. 2000; Tilson 1998).

Given that the most direct effect of agricultural practices is likely to be among farmers, agricultural workers, and their families, there is clearly potential as well for an impact on the surrounding communities, particularly among the children of those communities. To investigate that potential, we evaluated whether residence in a county with greater agricultural activity, as determined by percent of total land devoted to crop production, was associated with an increased risk of developing childhood cancers.

## Materials and Methods

### Cancer incidence and population data

Incidence data for U.S. children 0–14 years of age diagnosed with cancer between 1995 and 2001 were provided by member registries of the North American Association of Central Cancer Registries (NAACCR). To be eligible for the study, U.S. member registries had to agree to participate and had to have met at least NAACCR Silver certification requirements (NAACCR 2007). Given these criteria, 30 U.S. population-based state registries were initially eligible to be included in the analyses. County-level sex- and age-specific rates for the entire time period were obtained for all International Classification for Childhood Cancer (ICCC) site codes using the Surveillance Research Program, National Cancer Institute SEER*Stat software (SEER 2007) version 5.3.0. The National Cancer Institute receives population estimates from the U.S. Census Bureau’s Population Estimates Program through an interagency agreement. Accurate population data for Hawaii at the county level were not available, and Hawaiian counties were consequently excluded from the study population, leaving 29 eligible registries.

### County acreage, percent cropland, and percent acres in specific crops data

Data on the total land area by county in 2000 came from the database of county information maintained by the National Association of Counties (NACo), an organization that provides an extensive line of services including legislative, research, technical, and public affairs assistance to all U.S. counties (NACo 2005). Data from the 1997 U.S. Department of Agriculture (USDA) National Agricultural Statistics Service (NASS) Census of Agriculture were used to obtain information on both the acres of cropland and acres of land planted in six leading U.S. crops (barley, corn, cotton, oats, soybean, and wheat) in each of the counties in the study population (USDA 1997). This periodic census is the most thorough source of U.S. agricultural data from the county, state, and national level. We chose the 1997 census because it was conducted at the approximate midpoint of our incidence data. Of the 29 eligible state registries originally available for this study, no county identifiers were available for three and no NASS data were available for one. Eight of the state cancer registries did not have data available for all years of the study period, so counties from those registries were included only for years for which data were available. In addition, for some states, agricultural census data or age-specific population data were not available for every county, so those counties also were excluded from the analysis (*n* = 149), leaving a total of 25 U.S. states and 1,078 counties for inclusion in the final analysis.

### Statistical analysis

We derived the percent cropland for each county by dividing total land used for crop production by total land in acres for that county. On the basis of natural breaks in the overall distribution of percent cropland in the study area, we assigned counties with a total percent cropland of < 20% percent to the referent category (*n* = 515). For the remaining counties, categories of medium (20% to < 60%; *n* = 318) and high (≥ 60%; *n* = 245) agricultural activity were created. For evaluation of risk associated with the six leading U.S. crops, counties were classified as “exposed” if that crop was grown in that county, regardless of the percent of the total cropland for the county, and exposed counties were compared with counties that had both no recorded acreage planted in that crop and cropland totaling < 20% of the total county area.

For total cancers and then specific ICCC cancer subgroups, we compared the incidence rates in counties with low potential pesticide exposure to those with medium to high exposure, based on the categories for our surrogate of percent cropland. We estimated odds ratios (ORs) and associated 95% confidence intervals (CIs) by logistic regression, with adjustment for age and sex at the county level based on Census data provided by the SEER*Stat software. We also evaluated the data using Poisson regression, but results were equivalent to the logistic regression, so only those risk estimates are presented. All models were run in the SAS System for Windows V8 (SAS Institute Inc., Cary, NC).

## Results

**Figure 1:**
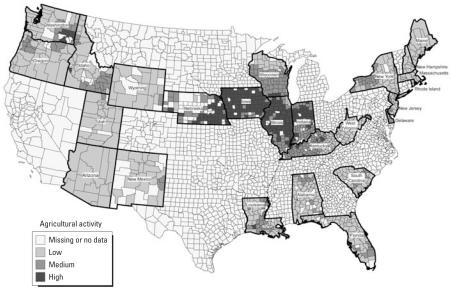
Location and category of agricultural activity for counties included in the study.

A total of 25 U.S. population-based cancer registries were included in the final study population (Figure 1), with a total average population at risk of 25,110,289 children 0–14 years of age (Table 1). The final sample included > 1,000 counties. The mean county size in acres was 670,572, and county size ranged from 15,796 acres in Rhode Island to 11,916,244 acres in Arizona. On average, approximately 32% of the total county acreage was cropland used for farming. There was quite a range of percent cropland among the study registries, with Arizona having the least average amount of cropland per county (1.8%) and Iowa averaging the most (74.7%).

There were slightly more males than females among both the at-risk population and the cancer cases (Table 2). The children in the at-risk population were approximately evenly divided among 0–4 (32%), 5–9 (34%), and 10–14 (34%) years of age. In contrast, cancer cases were younger, with 45% of the total cases being < 5 years of age. The distribution of the at-risk population and the total cancer cases, leukemia cases, and central nervous system cases by county percent cropland was approximately equivalent, with about 70% residing in counties with the lowest level of agricultural activity.

When only total cancers as a group were considered, no association was seen for percent cropland either for the medium or the high levels of agricultural activity (Table 3). In contrast, many specific cancer sites had statistically significantly elevated risk estimates for the medium agricultural activity category, including Hodgkin lymphoma (OR = 1.3; 95% CI, 1.1–1.5), Wilms’ tumor (OR = 1.3; 95% CI, 1.1–1.5), renal carcinomas (OR = 2.3; 95% CI, 1.3–4.2), hepatoblastoma (OR = 1.7; 95% CI, 1.3–2.3), Ewing’s sarcoma (OR = 1.8; 95% CI, 1.4–2.3), rhabdomyosarcomas (OR = 1.5; 95% CI, 1.2–1.7), thyroid carcinomas (OR = 1.8; 95% CI, 1.3–2.4), and malignant melanoma (OR = 1.6; 95% CI, 1.1–2.2). For the high exposure category (≥ 60% of the total county acreage devoted to farming), statistically significantly elevated ORs were seen for every cancer site examined, with many risk estimates showing two or more times the risk for childhood cancers when compared with the low level of agricultural activity. Additionally, there was a remarkably consistent indication of a possible dose–response effect when comparing risk estimates for the medium exposure category to the high.

As with the all-ages estimates, we observed no association at either exposure level for total childhood cancers when risk was examined separately for infants < 1 year of age or for 5-year age groups (i.e., 1–4, 5–9, and 10–14 years of age) (Table 4). Risk estimates for cancer subgroups generally mirrored that seen in the all-ages ORs, with statistically significantly increased risk seen predominantly in the high-exposure category for each age group. There generally was no clear pattern of risk associated with specific age groups for any of the individual cancers evaluated, perhaps except for neuroblastomas, where risk was elevated primarily for tumors diagnosed before 5 years of age, and lymphomas, osteosarcomas, and thyroid cancers, where risk was statistically significantly elevated only among the oldest ages. The majority of statistically significant ORs indicated a two-fold or greater risk for residence at diagnosis in exposed counties.

We observed a variety of patterns when we evaluated six individual crops for childhood cancer risk (Table 5). We found no statistically significant associations for any of the childhood cancers evaluated for residence at diagnosis in a county with barley crops, and only negative associations reached statistical significance for wheat production areas (hepatoblastoma, Ewing’s sarcoma, thyroid carcinomas, malignant melanomas, and other and unspecified cancers). Increased risk for cotton crops was seen only for renal carcinomas (OR = 6.9; 95% CI, 1.4–34.0). There were indications of increased risk for specific cancer sites with residence in counties planted in corn, oats, and soybeans. No childhood cancers had an increased risk that was associated with corn only. Those cancers associated with increased risk for oat crops only included primitive neuroectodermal tumors (OR = 1.5; 95% CI, 1.1–2.0), Ewing’s sarcoma (OR = 2.3; 95% CI, 1.4–3.7), germ cell tumors (OR = 2.6; 95% CI, 1.6–4.0), thyroid tumors (OR = 2.0; 95% CI, 1.2–3.4), and malignant melanoma (OR = 2.4; 95% CI, 1.3–4.5). Those cancers associated with increased risk for soybean production included only acute myeloid leukemias (OR = 1.4; 95% CI, 1.1–1.7), Hodgkin lymphoma (OR = 1.4; 95% CI, 1.1–1.8), and osteosarcoma (OR = 1.4; 95% CI, 1.1–1.9).

Several childhood cancers showed increased risk for pairs of crops. There was increased risk of non-Hodgkin lymphoma associated with residence at diagnosis in counties that produced either corn or oats, with the risk estimates equivalent for both crops (OR_corn_ = 1.5; 95% CI, 1.1–1.8; OR_oats_ = 1.5; 95% CI, 1.1–2.1). Thyroid cancer risk also was elevated for both corn- and oat-producing counties, again with the risk estimates roughly equivalent (OR_corn_ = 1.6; 95% CI, 1.1–2.3; OR_oats_ = 2.0; 95% CI, 1.2–3.4). Neuroblastomas and Wilms’ tumors showed increased risk for both corn (OR_neuroblastoma_ = 1.3; 95% CI, 1.1–1.5; OR_Wilm’s tumor_ = 1.4; 95% CI, 1.1–1.7) and soybean (OR_neuroblastoma_ = 1.3; 95% CI, 1.1–1.6; OR_Wilms’ tumor_ = 1.4; 95% CI, 1.1–1.7) production areas. Again, the risk estimates for the individual cancers were equivalent for both crops. Last, retinoblastoma risk was increased for both oats (OR = 1.6; 95% CI, 1.1–2.3) and soybean (OR = 1.4; 95% CI, 1.1–1.8) crops.

Corn and soybeans were produced in sufficient quantities in our study area to allow for an evaluation of risk associated with residence at diagnosis in counties with > 50% of the total county cropland dedicated to growing one or the other compared with counties not growing that crop and having < 20% total cropland. In general, risk estimates increased, and more reached statistical significance with this more conservative definition of exposure (data not shown).

## Discussion

Our study results indicate an increased risk for many types of childhood cancers associated with residence at diagnosis in counties having a moderate to high level of agricultural activity, with a remarkably consistent dose–response effect seen for counties having ≥ 60% of the total county acreage devoted to farming. Further, the finding that patterns of risk for individual cancers varied by crop type suggests that the development of different childhood cancers is likely to be related to specific pesticides.

A variety of chemical classes are represented by the pesticides applied to the six crops evaluated (Table 6). These data are taken from the NASS Agricultural Chemical Use Database (USDA 2006). Five of the six selected crops had one or more agricultural chemicals applied that have been designated by the U.S. Environmental Protection Agency as a possible carcinogen (U.S. EPA 2004). Very few epidemiologic studies have been able to evaluate cancer risk in general and childhood cancer risk in particular for specific agricultural chemicals. In two studies based in California, Reynolds et al. (2002, 2005) used information from California’s Department of Pesticide Regulation to examine risk associated with individual pesticides. The authors reported that neither analysis (one ecologic and one case–control) found consistent patterns of elevated risk for specific pesticides nor for classes of pesticides; however, only total cancers, leukemias, and central nervous system tumors were analyzed. Many of our more striking increased risk estimates were seen for cancers other than these leading types.

Epidemiologic studies have linked pesticide exposure to increased risk of several kinds of childhood cancers, generally through measurement of parental occupational exposures and/or residential pesticide use. Childhood leukemias and central nervous system tumors have been studied most extensively, perhaps because they are the more common types of what is a relatively rare disease, so the bulk of the epidemiologic evidence for a pesticide risk to children relates to these cancers. As noted in recent reviews, although study results have been mixed, overall this association has been most consistent for leukemias (Nasterlack 2006; Zahm and Ward 1998). Various studies have reported an elevated risk of brain tumors in farmers, with a recent meta-analysis finding an overall OR of 1.30 (95% CI, 1.09–1.56) for brain cancer and farming (Khuder et al. 1998). Several studies of farm-related exposures among pregnant mothers and their children have reported a parallel increase in risk for childhood brain tumors (Bunin et al. 1994; Cordier et al. 1994; Efird et al. 2003; Holly et al. 1998). Speculation about farm-related exposures of interest for childhood brain tumors has centered largely on agricultural pesticides and on farm animals [as a surrogate for an undetermined viral agent(s)].

Among the lymphomas, epidemiologic studies of risk associated with pesticide exposures have largely focused on non-Hodgkin lymphoma, and predominantly for cases diagnosed in adults. In studies evaluating non-Hodgkin lymphoma diagnosed among children, Zahm and Ward (1998) noted that several reported an apparent dose response to both agricultural and residential pesticide exposures.

The results for the few studies evaluating the possible risk associated with pesticide use and neuroblastomas in children have been equivocal; however, there is some evidence for an association for both occupational pesticide exposure of the parents and residential pesticide exposure of the family, particularly in studies that used specific pesticide exposure information rather than relying on parent’s job title (Daniels et al. 2001; Kristensen et al. 1996).

Retinoblastoma is a very low-incidence childhood tumor. There are two recognized types of retinoblastoma: one linked to genetic mutations and the other related to sporadic tumors. The heritable forms of retinoblastoma tend to be bilateral and occur during the first year of life. The sporadic nonheritable form is more likely to be unilateral and diagnosed after the first year of life (Ries et al. 1999). Had the risk been confined to children ≥ 1 year of age, this would have indicated that any putative association with agricultural pesticides is most relevant to the sporadic form; however, we saw increased risk estimates for the group < 1 year of age and up through 9 years of age.

We also found a statistically significant association in this study for malignant melanoma. Reports indicate that incidence of this cancer has been increasing among children and adolescents in recent decades (Hamre et al. 2002; Strouse et al. 2005). Sun exposure (both intermittent and total accumulated) and number of melanocytic and dysplastic nevi are well-established risk factors for malignant melanoma in adults (Armstrong and English 1996) and also appear to be related to risk in children (Strouse et al. 2005). Many of the exposed counties in the study area were located in more northerly states not normally associated with prolonged, intense sunlight exposure, so it seems unlikely that the increased risk would be attributed primarily to sun exposure. There has been an inconsistent pattern seen for melanoma risk associated with farmers and farming. Settimi et al. (1999, 2001) found an increased risk of melanoma in Italian farmers, but only among females. Another mortality study reported statistically significant lowered mortality risk for melanoma among Wisconsin farmers (Hanrahan et al. 1996). Interestingly, a cancer mortality study among farmers in Iowa reported an increased mortality risk for melanoma, but only among younger farmers (20–64 years of age) (Cerhan et al. 1998). There has been some speculation that insecticides in particular may have a link with development of malignant melanoma, possibly by affecting melanocytic function (Burkhart and Burkhart 2000).

Very little is known about the etiology of renal carcinomas, but there has been some indication of increased risk of Wilms’ tumor, the most common type of renal tumor in childhood, associated with possible occupational pesticide exposures and home applications (Sharpe et al. 1995; Zahm and Ward 1998). Similarly, because of the very few existing studies, there is little evidence available to evaluate the potential for an association between pesticide exposures and risk of some of the rarer childhood cancers we evaluated, including soft-tissue sarcomas, malignant bone tumors, germ cell tumors, and hepatic tumors (Nasterlack 2006; Zahm and Ward 1998).

Several limitations to our approach must be considered when interpreting the data. The exposure variable used is an imprecise surrogate for agriculturally related chemical exposures. However, of the 563 counties we categorized as exposed using this surrogate (i.e., having ≥ 20% of their total acreage in cropland), 332 (59%) had > 50% of their total county acreage in cropland, and 124 (22%) had a full three-quarters or more of their total acreage in agricultural production. In contrast, of the 515 referent counties, 357 (69%) had < 10% of their total acreage in cropland, and 224 (43%) had < 5% in cropland. These distributions illustrate the heterogeneity of possible exposure across the study area and lend support to our key assumption that children residing in an exposed county had a higher probability of encountering agricultural pesticides through pesticide drift than did the children residing in a referent county. Still, because there were so few counties with no agricultural activity, our unexposed population did include counties with up to 20% of total acreage in crop production as well as those counties with no farming, leaving the potential for misclassification of the exposure. This misclassification would move the risk estimates toward the null, though, and is unlikely to have generated the magnitude of risk seen for most cancer sites.

Additionally, we acknowledge there are a variety of ways it would be feasible to use existing agricultural data to attempt to capture any crop-specific effects, each with slightly different advantages and disadvantages. We believe our approach is valid to address the very general question of whether risk of individual cancer types varied according to probable differences in pesticides used, as defined by different crops grown. We evaluated this specifically because we saw uniformly increased risk across all cancer types when considering the main effect of percent cropland. Epidemiologic case–control studies, in contrast to this ecologic study, would be better suited for creating more specific exposure definitions based on cropping patterns and could better evaluate questions of dose–response and exposure timing for any specific pesticide (or pesticide surrogate).

We deliberately chose not to use existing urban/rural classification systems such as Rural–Urban Continuum Codes (RUCC; previously termed Beale codes) or Urban Influence Codes for this analysis because these systems are based largely on economic or population density measures, not agricultural production. Our classification approach was chosen specifically to capture density of agricultural activity at the county level. To evaluate the effectiveness of this approach, we compared our percent cropland classification with the RUCC for metropolitan (metro) and non-metropolitan areas (nonmetro) in our data. In this comparison, we found that of the 752 counties in our referent (i.e., low percentage of cropland and presumably “urban”) category, 432 (57.4%) were classified as nonmetro by RUCC coding. Further, we found that of the 292 counties classified by us as high percentage of cropland, 77 (26.4%) were classified as metro. Clearly, these data indicate that our analysis is not equivalent to a standard “urban” versus “rural” comparison.

Use of county of residence at time of diagnosis may be considered another limitation of this study. If the exposures of interest are most pertinent during gestation, then, if available, mother’s county of residence at time of birth or time of conception would be the preferred measure for assessing the impact of exposure to agricultural chemicals. Because pesticides can act as either initiators or promoters, however, it is plausible that some pesticides may influence cancer development nearer to time of diagnosis.

We also had very few data available to address any potential confounding, always a concern in epidemiologic studies. The evidence for most putative risk factors for the different childhood cancers is conflicting, so any effect from potential confounders is likely to be weak, particularly when dispersed across the county, our unit of analysis, and when many different types of cancers are considered in the analysis, as in our study. Although there might be a specific concern about differential use of residential pesticides in the populations living in counties with low agricultural activity (i.e., more urban counties) versus those with medium or high agricultural activity (i.e., more rural), published reports agree that there is very little difference in household use of pesticides in urban versus nonurban settings (Adgate et al. 2000; U.S. EPA 2007)

In addition to general concerns regarding the ability to determine causality that apply to any ecologic study, our approach requires several assumptions, including *a*) that mobility of study subjects is not sufficient to substantially affect risk estimates, and *b*) that the cropland data derived from the 2 years of agricultural census information are consistent across the study years. Finally, we have no ready explanation for the lack of an effect seen when evaluating all cancer types together compared with our results for individual cancer types. Because the OR is not a linear transformation of these data, we cannot expect that the OR for all cancers would be the average of the ORs for the subgroups. It is possible that we may have experienced some form of Simpson’s paradox in our data set when combining the cancer types into one “super” group (Simpson 1951).

The most notable strength of this study is the large number of counties included. This large sample size gave us the ability to evaluate rarer childhood cancers and resulted in stable risk estimates. In addition, the sample constituted a geographically diverse area and included states that produce a variety of crops, enabling us to evaluate whether risk differed by crop type.

Our finding of statistically significant increased risk across all cancer types evaluated for those counties having ≥ 60% of total acreage in cropland was unexpected and, given the ecologic design of the study, needs to be interpreted with considerable caution. Several factors, however, argue against this finding being an artifact of the data or the data analysis approach chosen. There was a consistent dose–response relationship seen between risk estimates for our medium- compared with our high-exposure categories. In contrast to the general focus in the epidemiologic literature on the more common childhood cancers, we were able to evaluate rarer childhood cancers. The growing regions for the different crops generally did not overlap, so it is unlikely that we simply captured a high-risk population. The patterns of risk varied according to crop type. Taken together, these features of the study indicate the potential for a relationship between pesticides, or at least agricultural activity in general, and childhood cancers, with the magnitude of the risk possibly being two or more times that of nonfarming areas.

The biological mechanisms by which pesticides may be involved in childhood cancers include acting as initiators (i.e., mutagens) or tumor promoters, affecting immune system regulation, or possibly through mimicking estrogen or otherwise disrupting endogenous hormonal activity (Dich et al. 1997). Although it seems unlikely that any one pesticide would result in the range of risks reported in this study, it does seem plausible that many different pesticides, acting through a variety of mechanisms, could be linked to a variety of childhood cancers.

This study is meant to provide an alternative look at the possible impact of agricultural practices on cancer risk in surrounding communities, with our method being particularly amenable to childhood cancers, because these cancers have a much shorter latency period than do adult cancers. With data accumulating regarding the atmospheric transport of pesticides over long distances (van den Berg et al. 1999) and reports indicating that some level of pesticide exposure is nearly ubiquitous in the U.S. population (CDC 2005), it is likely that there will continue to be interest in the possible impact of long-term, low-level pesticide exposure in human populations, particularly among infants and young children.

## Figures and Tables

**Table 1:** Characteristics of the population-based U.S. cancer registries included in the study, NAACCR, 1995–2001.

				County size (acres)				Percent county cropland No.^c^	
State (data years)	Population at risk (no.)^a^	Childhood cancer incidence rate^b^	SE	No.	Mean	Minimum	Maximum	No.^c^	Mean ± SD
Alabama (1998–2001)	923,182	108.6	5.4	67	484,778	342,296	1,021,780	57	13.0 ± 8.0
Arizona (1995–2001)	1,093,842	128.8	4.1	15	4,848,735	792,158	11,916,244	10	1.8 ± 2.3
Delaware (1997–2000)	158,387	155.1	15.7	3	416,985	272,831	600,101	3	37.0 ± 11.0
Florida (1995–2001)	2,990,616	148.9	2.7	67	515,795	153,791	1,301,974	60	10.3 ± 9.1
Idaho (1995–2001)	295,656	147.8	8.5	44	1,203,650	260,816	5,430,522	37	20.0 ± 16.7
Illinois (1995–2001)	2,686,668	144.8	2.8	102	348,821	102,274	757,530	95	66.6 ± 17.5
Indiana (1998–2001)	1,306,617	142.7	5.2	92	249,531	55,508	420,662	83	56.6 ± 18.7
Iowa (1995–2001)	592,570	158.2	6.2	99	361,211	243,884	622,766	91	74.7 ± 11.0
Kentucky (1995–2001)	809,827	147.1	5.1	120	211,906	63,258	504,145	109	34.6 ± 20.9
Louisiana (1995–2001)	1,009,052	116.6	4.1	64	435,661	115,616	850,245	60	19.9 ± 19.0
Maine (1995–2001)	246,630	173.8	10.2	16	1,234,581	162,566	4,270,039	16	3.6 ± 2.3
Massachusetts (1997–2001)	1,244,435	152.8	5.0	14	358,306	30,580	968,424	12	4.2 ± 2.1
Nebraska (1995–2001)	367,540	128.7	7.1	93	529,051	154,030	3,814,878	58	63.6 ± 19.1
New Hampshire (1999–2001)	255,944	120.4	12.7	10	574,040	236,051	1,152,415	10	2.6 ± 1.0
New Jersey (1995–2001)	1,728,136	155.9	3.6	21	235,910	66,104	515,052	20	11.6 ± 10.3
New Mexico (1995–2001)	419,775	127.1	6.6	33	2,353,736	69,987	4,434,092	23	5.3 ± 10.8
New York (1997–2001)	3,891,864	154.4	2.8	62	495,022	18,162	1,718,863	57	17.8 ± 13.0
Oregon (1996–2001)	692,795	146.8	6.0	36	1,706,711	278,570	6,486,330	34	10.5 ± 10.7
Rhode Island (1995–2001)	203,255	180.4	11.3	5	133,759	15,796	264,506	5	5.5 ± 4.0
South Carolina (1997–2001)	827,867	129.3	5.6	46	418,937	230,140	725,577	43	13.6 ± 8.3
Utah (1995–2001)	577,001	142.2	5.9	29	1,813,366	194,875	5,005,263	25	5.6 ± 5.4
Washington (1995–2001)	1,232,017	157.8	4.3	39	1,092,615	111,963	3,371,698	34	17.0 ± 23.2
West Virginia (1995–2001)	333,797	133.7	7.6	55	280,280	53,149	665,470	50	9.9 ± 7.2
Wisconsin (1995–2001)	1,117,019	148.6	4.4	72	482,788	148,456	988,848	68	34.4 ± 18.5
Wyoming (1995–2001)	105,796	156.0	14.7	23	2,702,040	1,282,556	6,672,552	18	5.7 ± 4.8
Total	25,110,289	145.5	1.0	1,227	670,572	15,796	11,916,244	1,078	32.3 ± 28.3

a Average annual population at risk, children 0–14 years of age.

b Rates are average annual per 100,000,000 population for 0–14 years of age and are age-adjusted to the 2000 U.S. standard million population.

c Differences in county numbers are attributed to missing agricultural census data or age-specific population data.

**Table 2:** Distribution of total childhood, leukemia, and central nervous system (CNS) cancer cases and at-risk population by sex, age, and percent cropland for study area, NAACCR 1995–2001.

Characteristic	At-risk population [no. (%)]	All cancers [cases (%)]	Leukemia [cases (%)]	CNS [cases (%)]
Sex				
Male	71,321,275 (51)	10,856 (54)	3,354 (54)	2,334 (54)
Female	67,914,027 (49)	9,378 (46)	2,814 (46)	1,984 (46)
Age group (years)				
0	8,838,525 (6)	2,095 (10)	321 (5)	289 (7)
1–4	35,861,584 (26)	7,123 (35)	2,944 (48)	1,310 (30)
5–9	47,188,891 (34)	5,195 (26)	1,689 (27)	1,491 (35)
10–14	47,346,302 (34)	5,821 (29)	1,214 (20)	1,228 (28)
County percent cropland				
< 20	97,330,441 (70)	14,160 (70)	4,341 (70)	2,995 (69)
20 to < 60	31,217,835 (22)	4,476 (22)	1,357 (22)	975 (23)
≥ 60	10,687,026 (8)	1,598 (8)	470 (8)	348 (8)

**Table 3:** Estimated ORs (95% CIs) by age groups for childhood cancers associated with residence at diagnosis in agriculturally intense counties in the U.S. NAACCR, 1995–2001, ICCC major sites and selected subgroups.

	Percent cropland			
	Medium		High	
Cancer type	No.	OR (95% CI)	No.	OR (95% CI
All cancers	4,476	1.0 (1.0–1.0)	1,598	1.0 (1.0–1.1)
Leukemias	1,357	1.0 (0.9–1.1)	470	1.2 (1.1–1.3)
Lymphoid leukemias	1,074	1.0 (0.9–1.1)	387	1.3 (1.1–1.4)
Acute myeloid leukemias	207	1.2 (1.1–1.4)	66	1.8 (1.4–2.3)
Lymphomas and reticuloendothelial	463	1.1 (1.0–1.2)	155	1.4 (1.2–1.7)
Hodgkin lymphomas	189	1.3 (1.1–1.5)	58	2.1 (1.6–2.7)
Non-Hodgkin lymphomas	166	1.1 (0.9–1.3)	60	2.1 (1.6–2.8)
Central nervous system	975	1.1 (1.0–1.1)	348	1.3 (1.1–1.4)
Astrocytomas	475	1.1 (1.0–1.2)	184	1.5 (1.3–1.7)
Primitive neuroectodermal tumor	222	1.2 (1.0–1.4)	69	1.9 (1.5–2.4)
Sympathetic nervous system tumors	349	1.2 (1.0–1.3)	121	1.7 (1.4–2.1)
Neuroblastomas	336	1.2 (1.0–1.3)	118	1.8 (1.5–2.1)
Retinoblastoma	97	1.2 (1.0–1.5)	45	2.6 (1.9–3.5)
Renal tumors	269	1.3 (1.1–1.5)	102	2.1 (1.7–2.6)
Wilms’ tumor	253	1.3 (1.1–1.5)	96	2.1 (1.7–2.7)
Renal carcinoma	16	2.3 (1.3–4.2)	5	3.3 (1.3–8.3)
Hepatic tumors	63	1.6 (1.2–2.2)	23	3.3 (2.1–5.0)
Hepatoblastoma	54	1.7 (1.3–2.3)	21	4.0 (2.5–6.3)
Malignant bone tumors	218	1.2 (1.1–1.4)	84	2.3 (1.8–2.9)
Osteosarcoma	110	1.3 (1.0–1.6)	44	2.7 (2.0–3.6)
Ewing’s sarcoma	91	1.8 (1.4–2.3)	33	4.3 (3.0–6.2)
Soft-tissue sarcomas	344	1.3 (1.1–1.4)	109	1.7 (1.4–2.0)
Rhabdomyosarcomas	181	1.5 (1.2–1.7)	59	2.5 (1.9–3.3)
Germ cell, etc.^a^	130	1.3 (1.1–1.6)	57	2.3 (1.8–3.1)
Carcinomas and other	164	1.1 (1.0–1.3)	77	2.2 (1.8–2.8)
Thyroid carcinoma	55	1.8 (1.3–2.4)	26	3.0 (2.0–4.6)
Malignant melanoma	41	1.6 (1.1–2.2)	25	4.6 (3.0–7.0)
Other and unspecified	25	2.9 (1.9–4.6)	7	11.2 (5.1–24.4)

a Germ cell, trophoblastic, and other gonadal neoplasms.

**Table 4:** Estimated ORs (95% CIs) by age groups for childhood cancers associated with residence at diagnosis in agriculturally intense counties in the U.S. NAACCR, 1995–2001, ICCC major sites and selected subgroups.

	Age 0		Age 1–4		Age 5–9		Age 10–14	
Cancer type	Medium^a^	High^b^	Medium^a^	High^b^	Medium^a^	High^b^	Medium^a^	High^b^
All cancers	0.9 (0.8–1.0)	1.0 (0.8–1.1)	1.0 (1.0–1.1)	1.1 (1.0–1.2)	1.0 (0.9–1.1)	1.0 (0.9–1.1)	1.0 (0.9–1.0)	1.1 (1.0–1.2)
Leukemias	1.0 (0.8–1.3)	0.6 (0.3–1.1)	1.0 (0.9–1.1)	1.3 (1.1–1.5)	1.0 (0.9–1.1)	1.1 (0.9–1.3)	0.9 (0.8–1.1)	1.1 (0.9–1.4)
Lymphoid leukemias	1.1 (0.8–1.6)	0.4 (0.1–1.0)	1.0 (0.9–1.1)	1.4 (1.2–1.6)	1.0 (0.8–1.1)	1.1 (0.9–1.4)	1.0 (0.8–1.1)	1.3 (1.1–1.7)
Acute myeloid leukemias	1.1 (0.7–1.7)	1.6 (0.7–3.5)	1.1 (0.9–1.5)	2.1 (1.4–3.2)	1.6 (1.1–2.2)	1.7 (0.9–3.0)	1.2 (0.9–1.5)	1.6 (1.0–2.6)
Lymphomas	1.2 (0.7–2.3)	1.9 (0.7–4.7)	1.2 (1.0–1.6)	1.1 (0.7–1.9)	1.0 (0.8–1.2)	1.2 (0.8–1.6)	1.1 (0.9–1.2)	1.6 (1.3–2.0)
Hodgkin lymphoma	2.6 (0.4–15.7)	—	1.7 (0.8–3.5)	0.8 (0.1–6.0)	1.2 (0.8–1.7)	1.7 (0.9–3.2)	1.3 (1.1–1.6)	2.2 (1.7–3.0)
Non-Hodgkin lymphoma	1.2 (0.4–3.7)	3.2 (0.7–14.1)	1.4 (0.9–2.0)	1.9 (1.0–3.7)	1.1 (0.8–1.5)	1.7 (1.0–2.8)	1.0 (0.8–1.3)	2.5 (1.7–3.6)
Central nervous system	1.1 (0.8–1.4)	1.0 (0.7–1.7)	1.0 (0.9–1.2)	1.4 (1.2–1.7)	1.0 (0.9–1.1)	1.1 (0.9–1.4)	1.2 (1.0–1.3)	1.3 (1.1–1.6)
Astrocytomas	0.7 (0.5–1.2)	1.0 (0.5–2.0)	1.1 (0.9–1.3)	1.5 (1.1–2.0)	1.1 (0.9–1.3)	1.4 (1.0–1.8)	1.2 (1.0–1.4)	1.7 (1.4–2.2)
PNET	1.6 (1.0–2.7)	2.2 (1.0–5.1)	1.2 (0.9–1.5)	2.3 (1.5–3.4)	1.1 (0.9–1.4)	1.7 (1.2–2.6)	1.3 (0.9–1.7)	1.6 (0.9–2.7)
Sympathetic nervous system	1.0 (0.8–1.3)	1.8 (1.3–2.5)	1.2 (1.0–1.4)	1.8 (1.4–2.4)	1.3 (0.9–1.9)	1.2 (0.6–2.3)	1.3 (0.7–2.3)	1.3 (0.5–3.6)
Neuroblastomas	1.0 (0.8–1.3)	1.9 (1.4–2.5)	1.2 (1.0–1.4)	1.8 (1.4–2.4)	1.4 (0.9–1.9)	1.3 (0.7–2.6)	1.2 (0.6–2.4)	0.9 (0.2–3.7)
Retinoblastoma	1.3 (0.9–1.8)	2.6 (1.6–4.2)	1.2 (0.9–1.6)	2.3 (1.5–3.6)	1.1 (0.4–3.3)	6.7 (2.5–18.1)	—	—
Renal tumors	1.4 (1.0–2.0)	2.7 (1.7–4.5)	1.3 (1.1–1.5)	2.1 (1.6–2.7)	1.3 (1.0–1.8)	1.8 (1.1–3.0)	1.5 (0.9–2.6)	2.3 (1.0–5.3)
Wilms’ tumor	1.5 (1.0–2.1)	2.5 (1.4–4.2)	1.3 (1.1–1.6)	2.1 (1.6–2.8)	1.3 (0.9–1.7)	1.9 (1.2–3.1)	1.5 (0.7–3.5)	2.4 (0.7–8.1)
Renal carcinoma	0.8 (0.1–6.6)	7.4 (1.5–35.5)	1.9 (0.2–18.1)	—	4.6 (1.4–15.2)	—	2.2 (1.0–4.8)	3.3 (1.0–11.1)
Hepatic tumors	1.7 (1.1–2.8)	2.1 (0.9–5.3)	1.6 (1.0–2.4)	4.6 (2.7–8.0)	2.0 (0.8–4.7)	4.7 (1.4–16.1)	1.3 (0.5–3.2)	—
Hepatoblastoma	1.7 (1.0–2.8)	2.4 (1.0–6.0)	1.7 (1.1–2.6)	4.7 (2.6–8.5)	2.3 (0.7–7.5)	10.6 (2.9–39.3)	1.0 (0.1–8.7)	—
Malignant bone tumors	—	6.1 (0.6–58.4)	1.7 (0.9–3.2)	0.6 (0.1–4.0)	1.5 (1.1–2.0)	3.0 (2.0–4.5)	1.1 (0.9–1.4)	2.1 (1.6–2.8)
Osteosarcoma	—	—	2.7 (0.9–8.4)	—	1.5 (1.0–2.2)	2.1 (1.0–4.2)	1.2 (0.9–1.5)	3.0 (2.1–4.2)
Ewing’s sarcoma	—	34.6 (2.2–553.3)	2.3 (1.1–4.7)	1.5 (0.2–11.0)	2.3 (1.5–3.6)	7.4 (4.2–13.3)	1.6 (1.1–2.1)	3.3 (2.0–5.5)
Soft-tissue sarcomas	1.1 (0.8–1.7)	1.5 (0.8–2.9)	1.2 (0.9–1.5)	1.6 (1.1–2.3)	1.5 (1.2–1.9)	2.1 (1.4–2.9)	1.2 (1.0–1.5)	1.5 (1.0–2.1)
Rhabdomyosarcomas	1.3 (0.7–2.4)	2.5 (1.0–6.4)	1.3 (1.0–1.7)	2.6 (1.7–3.9)	1.6 (1.2–2.2)	2.8 (1.7–4.7)	1.7 (1.2–2.3)	2.0 (1.1–3.6)
Germ cell, etc.^c^	1.1 (0.7–1.8)	2.5 (1.3–4.5)	1.7 (1.1–2.6)	0.9 (0.3–2.5)	1.3 (0.8–2.1)	3.3 (1.8–6.0)	1.2 (0.9–1.6)	2.5 (1.7–3.7)
Carcinomas and other^d^	0.6 (0.3–1.4)	0.8 (0.2–3.1)	1.2 (0.6–2.3)	0.9 (0.2–3.8)	1.2 (0.8–1.8)	1.2 (0.6–2.6)	1.2 (1.0–1.4)	2.8 (2.1–3.6)
Thyroid carcinoma	6.1 (0.9–43.6)	—	1.0 (0.1–8.4)	—	1.8 (0.9–3.6)	1.8 (0.6–6.0)	1.7 (1.2–2.4)	3.5 (2.3–5.4)
Malignant melanoma	1.0 (0.2–4.6)	—	3.3 (1.3–8.2)	4.6 (1.0–20.1)	1.1 (0.4–2.7)	2.1 (0.5–8.6)	1.6 (1.0–2.4)	5.9 (3.7–9.3)
Other and unspecified	2.4 (0.8–7.3)	24.5 (7.1–85.4)	5.9 (2.7–12.8)	—	4.8 (2.0–11.6)	16.6 (3.7–73.5)	0.6 (0.1–2.5)	7.9 (1.9–33.3)

Abbreviations: —, no cases; PNET, primitive neuroectodermal tumor.

a 20% to < 60% of total county acreage in cropland.

b ≥ 60% of total county acreage in cropland.

c Germ cell, trophoblastic, and other gonadal neoplasms.

d Carcinomas and other malignant epithelial neoplasms.

**Table 5:** Estimated ORs^a^ (95% CIs) for childhood cancers associated with specific crops, NAACCR, 1995–2001, ICCC major sites and selected subgroups.

Cancer type	Barley	Corn	Cotton	Oats	Soybean	Wheat
All cancers	0.9 (0.8–1.0)	1.0 (0.9–1.0)	1.0 (0.9–1.1)	1.0 (0.9–1.1)	1.0 (1.0–1.1)	1.1 (1.0–1.2)
Leukemias	1.0 (0.8–1.2)	1.0 (0.9–1.1)	1.1 (1.0–1.3)	1.1 (1.0–1.3)	1.0 (0.9–1.1)	1.0 (0.9–1.2)
Lymphoid leukemias	1.0 (0.8–1.2)	1.0 (0.9–1.1)	1.1 (0.9–1.3)	1.2 (1.0–1.4)	1.0 (0.9–1.0)	1.0 (0.9–1.1)
Acute myeloid leukemias	1.0 (0.6–1.5)	1.2 (1.0–1.5)	1.0 (0.7–1.5)	1.1 (0.8–1.5)	1.4 (1.1–1.7)	0.9 (0.6–1.2)
Lymphomas and reticuloendothelial	1.1 (0.8–1.4)	1.2 (1.0–1.4)	1.0 (0.8–1.3)	1.2 (1.0–1.4)	1.2 (1.1–1.4)	1.0 (0.8–1.3)
Hodgkin lymphomas	1.0 (0.6–1.6)	1.2 (1.0–1.5)	0.8 (0.5–1.2)	1.2 (0.9–1.7)	1.4 (1.1–1.8)	1.1 (0.7–1.6)
Non-Hodgkin lymphomas	1.0 (0.6–1.5)	1.5 (1.1–1.8)	0.9 (0.6–1.3)	1.5 (1.1–2.1)	1.3 (1.0–1.7)	0.8 (0.6–1.1)
Central nervous system	0.8 (0.7–1.0)	1.0 (0.9–1.1)	0.9 (0.7–1.1)	1.1 (1.0–1.3)	1.0 (0.9–1.1)	1.1 (0.9–1.2)
Astrocytomas	0.7 (0.6–1.0)	1.1 (1.0–1.3)	0.8 (0.6–1.1)	1.3 (1.0–1.6)	1.0 (0.9–1.2)	0.9 (0.8–1.2)
PNET	0.8 (0.6–1.1)	1.1 (0.9–1.3)	0.8 (0.6–1.2)	1.5 (1.1–2.0)	1.2 (1.0–1.5)	0.9 (0.6–1.1)
Sympathetic nervous system tumors	0.8 (0.6–1.1)	1.3 (1.1–1.5)	1.0 (0.7–1.4)	1.1 (0.9–1.4)	1.3 (1.1–1.6)	0.9 (0.7–1.2)
Neuroblastomas	0.8 (0.6–1.1)	1.3 (1.1–1.5)	1.0 (0.7–1.3)	1.1 (0.9–1.4)	1.3 (1.1–1.6)	0.9 (0.7–1.2)
Retinoblastoma	0.9 (0.6–1.6)	1.2 (0.9–1.5)	1.0 (0.6–1.7)	1.6 (1.1–2.3)	1.4 (1.1–1.8)	0.6 (0.4–1.0)
Renal tumors	0.9 (0.6–1.3)	1.3 (1.1–1.6)	0.8 (0.6–1.1)	1.0 (0.8–1.3)	1.4 (1.2–1.7)	0.9 (0.6–1.1)
Wilms’ tumor	0.9 (0.6–1.4)	1.4 (1.1–1.7)	0.8 (0.6–1.1)	1.1 (0.8–1.4)	1.4 (1.1–1.7)	0.8 (0.6–1.1)
Renal carcinoma	4.7 (0.7–33.3)	1.2 (0.6–2.6)	6.9 (1.4–34.0)	2.2 (0.8–5.7)	2.0 (0.8–5.0)	0.3 (0.0–2.0)
Hepatic tumors	0.6 (0.3–1.0)	1.2 (0.8–1.7)	0.6 (0.3–1.3)	1.4 (0.9–2.3)	1.1 (0.8–1.7)	0.6 (0.3–1.2)
Hepatoblastoma	0.6 (0.3–1.1)	1.3 (0.9–1.9)	0.5 (0.2–1.1)	1.7 (1.0–2.8)	1.1 (0.7–1.7)	0.4 (0.2–0.7)
Malignant bone tumors	1.3 (0.8–2.2)	1.2 (1.0–1.4)	0.8 (0.5–1.1)	1.3 (1.0–1.8)	1.2 (1.0–1.4)	0.9 (0.7–1.3)
Osteosarcoma	1.3 (0.7–2.5)	1.3 (1.0–1.7)	0.7 (0.5–1.2)	1.4 (0.9–2.0)	1.4 (1.1–1.9)	0.8 (0.6–1.2)
Ewing’s sarcoma	1.1 (0.5–2.4)	1.4 (1.0–2.0)	0.6 (0.3–1.2)	2.3 (1.4–3.7)	1.3 (1.0–1.8)	0.4 (0.3–0.7)
Soft-tissue sarcomas	0.9 (0.7–1.3)	1.1 (1.0–1.3)	0.9 (0.6–1.2)	1.2 (0.9–1.5)	1.2 (1.0–1.4)	0.9 (0.7–1.1)
Rhabdomyosarcomas	1.2 (0.7–2.0)	1.3 (1.0–1.6)	0.9 (0.6–1.5)	1.2 (0.9–1.7)	1.3 (1.0–1.7)	0.9 (0.6–1.3)
Germ cell, etc.^b^	0.7 (0.5–1.1)	1.3 (1.0–1.7)	1.0 (0.7–1.6)	2.6 (1.6–4.0)	1.2 (0.9–1.5)	1.1 (0.7–1.7)
Carcinomas and other^c^	1.0 (0.6–1.6)	1.1 (0.9–1.4)	1.1 (0.8–1.6)	1.3 (1.0–1.8)	1.3 (1.0–1.6)	0.9 (0.6–1.2)
Thyroid carcinoma	1.7 (0.6–4.9)	1.6 (1.1–2.3)	0.5 (0.2–1.3)	2.0 (1.2–3.4)	1.4 (1.0–2.1)	0.4 (0.3–0.7)
Malignant melanoma	1.3 (0.6–2.9)	1.3 (0.8–2.0)	0.6 (0.3–1.3)	2.4 (1.3–4.5)	1.2 (0.8–1.9)	0.4 (0.2–0.8)
Other and unspecified	1.1 (0.3–3.9)	1.6 (0.8–3.0)	0.2 (0.0–0.5)	3.2 (1.3–7.8)	1.0 (0.5–1.7)	0.3 (0.2–0.7)

PNET, primitive ectodermal tumor. Referent category is counties with no acreage in the specific crop being evaluated and total percent cropland < 20%.

a ORs adjusted for age and sex.

b Germ cell, trophoblastic, and other gonadal neoplasms.

c Carcinomas and other malignant epithelial neoplasms.

**Table 6:** Top five agricultural chemicals applied (by percent treated acres), by crop.^a^

Chemical	Percent of treated acres	Chemical class	Carcinogenic potential
Barley^b^			
2,4-D	35	Chlorphenoxy acid/ester	D-not classifiable
MCPA	22	Chlorphenoxy acid/ester	Not likely
Bromoxynil	14	Hydroxybenzonitrile	C-possible carcinogen
Tribenuron methyl	12	Sulfonylurea	C-possible carcinogen
Thifensulfuron	8	Sulfonylurea	Not listed
Corn^c^			
Atrazine	70	Triazine	Not likely
Metolachlor	24	Chloroacetanilide	C-possible carcinogen
Acetochlor	24	Chloroacetanilide	Likely carcinogen
Dicamba	21	Benzoic acid	D-not classifiable
Nicosulfuron	12	Sulfonylurea	E-evidence of noncarc
Cotton-upland^c^			
Trifluralin	50	2,6-Dinitroaniline	C-possible carcinogen
Ethephon	33	Organophosphate	D-not classifiable
Tribufos	31	Organophosphate	Likely at high dose only
Fluometuron	29	Urea	E-evidence of noncarc
Aldicarb	23	*N*-methyl carbamate	E-evidence of noncarc
Oats^b^			
2,4-D	13	Chlorphenoxy acid/ester	D-not classifiable
MCPA	6	Chlorphenoxy acid/ester	Not likely
Dicamba	3	Benzoic acid	D-not classifiable
Glyphosate	2	Phosphonoglycine	E-evidence of noncarc
Chlorsulfuron	2	Sulfonylurea	E-evidence of noncarc
Soybeans^c^			
Glyphosate	45	Phosphonoglycine	E-evidence of noncarc
Imazethapyr	26	Imidazolinone	E-evidence of noncarc
Pendimethalin	19	2,6-Dinitroaniline	C-possible carcinogen
Trifluralin	16	2,6-Dinitroaniline	C-possible carcinogen
Chlorimuron ethyl	12	Sulfonylurea	Not listed
Wheat (spring, winter)^d^			
2,4-D	38	Chlorphenoxy acid/ester	D-not classifiable
MCPA	24	Chlorphenoxy acid/ester	Not likely
Dicamba	17	Benzoic acid	D-not classifiable
Tribenuron methyl	13	Sulfonylurea	C-possible carcinogen
Fenoxyprop	12	Aryloxyphenoxy propionic acid	Not listed

Abbreviations: 2,4-D, 2,4-dichlorophenoxyacetic acid; MCPA, 4-chloro-2-methylphenoxyacetic acid; noncarc, noncarcinogenicity.

a Data from USDA (2006) and U.S. EPA (2004). Multiyear data are means.

b 1998 data only.

c 1995–2001 data.

d 1995–1998, 2000 data; durum wheat excluded because it is grown only in North Dakota.
